# Sense and Nonsense

**Published:** 1856-05

**Authors:** 


### SENSE AND NONSENSE.

Many persons have the intelligence to feel that exercise is essential to good health, but domestic and financial duties press upon them so much, that it is only occasionally that the claims of health attract their practical attention, and then they go about it with a kind of spasmodic desperation, as if they intended to do as much in a day as would answer for a month past and to come. The early spring time has a peculiar influence in waking up the dormant industries of this class of persons, and on some sunny morning they sally out with rake or axe, or spade or hoe, and with the energy of a *Quarter Horse*, they carry everything before them for an hour, or perhaps several hours, when before they are aware of it, their strength is exhausted, they feel "*weak as water*," the whole body is in a perspiration, and weary and worn out, and overheated, they make for the house, the ordinary warmth of which now seems oppressive, and with hat and coat, or shawl lain aside, they throw themselves on the sofa in some cool part of the house and fall asleep, or if they do not, they take early supper and go to bed, waking up in the morning haggard, sickish, and as stiff and sore in joint and limb and muscle, as a veteran Rheumatic of half a century; and for days, if not for weeks, they feel more dead than alive, and come to the conclusion that exercise does not agree with them, and it takes them about a year to get rid of the conviction.

For sedentary persons to exercise safely and with advantage, a few rules should be strictly adhered to.

1. Let your labor be moderate and of short duration for the first day, gradually increasing it from day to day in time and intensity.

2. The moment you cease the exercise, whatever it may be, put on the garments you laid aside before you began, go at once to the house and sit down by a fire or in some warm room or kitchen, if necessary, without washing, or drinking or eating, and in the course of fifteen minutes, according to circumstances, push back from the fire, take off your hat, next lay aside any surplus garment, then wash your face and hands in tepid, if not warm water, with soap, take a very light supper, that is, a piece of cold bread and butter, and half a glass of water, and at your usual hour retire to bed. Exercise, with such precautions, will seldom fail to yield the richest and most enduring results, a sound sleep for the night, a keen appetite in the morning, with a feeling of newness and freshness and vigor next day, delightful to think of.

We cannot here enter into a detailed explanation of the reasons for all this, but will merely state the governing idea, which is, that *getting cool slowly* makes all the difference between exercise which is beneficial and exercise which aggravates the evils it was intended to cure.

To impress this on the mind more fully, we have only to state this interesting fact, that on the surface of the body there are millions of little tubes which are always conveying effete, useless matter from the system, either in a solid, fluid, or gaseous form, but during exercise these operations are carried on with greatly increased activity; a dash of cold air or cold water instantly closes up the outlet of each one of these little tubes, which, if placed continuously, would amount to many miles in length, and this sudden check is as infallible a cause of bodily calamity as the explosion of a steam boiler under a full head of steam, if the valve is shut and kept down after the engine has ceased motion. Hence no man ever did, or ever can fall asleep uncovered, or in a draft of air after exercising, without waking up with unpleasant feelings of all degrees from a slight pain or soreness to the agonies of dissolution in a few hours.

How illy nature bears the sudden arrest of some of her operations, is strikingly exemplified in the fact that if the blandest of all liquids, lukewarm milk, is injected into a blood-vessel against the current, instant death may result, but if introduced gently in the direction of the current, it is borne with impunity.

